# (3*S*,7a*R*)-7-Meth­oxy-7a-methyl-3-phenyl-2,3-dihydro­pyrrolo[2,1-*b*]oxazol-5(7a*H*)-one

**DOI:** 10.1107/S1600536808040105

**Published:** 2008-12-06

**Authors:** Jian-Feng Zheng, Li-Jiao Jiang, Jian-Liang Ye

**Affiliations:** a The Key Laboratory for Chemical Biology of Fujian Province, College of Chemistry and Chemical Engineering, Xiamen University, Xiamen, Fujian 361005, People’s Republic of China

## Abstract

In the title chiral butterfly-like bicyclic lactam, C_14_H_15_NO_3_, the phenyl and methyl groups are *syn* with respect to each other. The dihydro­pyrrrole ring adopts a boat conformation, whereas the oxazole ring has a slightly distorted boat conformation. The packing of mol­ecules in the crystal structure is stabilized by inter­molecular C—H⋯O hydrogen bonds.

## Related literature

For reference bond-length data, see: Allen *et al.* (1987 ▶). For the chemistry of tetra­mic acids and methyl tetra­mates, see: Huang & Deng (2004 ▶); Huang *et al.* (2003 ▶); Jiang *et al.* (2009 ▶).
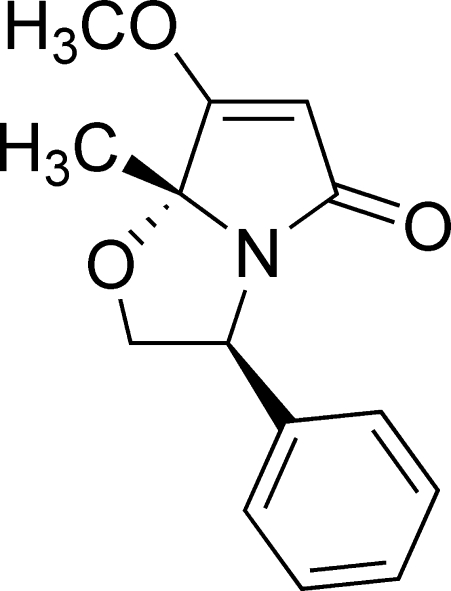


## Experimental

### 

#### Crystal data

C_14_H_15_NO_3_
*M* *_r_* = 245.27
Monoclinic, 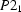

*a* = 7.8238 (10) Å
*b* = 5.9033 (7) Å
*c* = 13.711 (3) Å
β = 96.597 (14)°
*V* = 629.05 (16) Å^3^
*Z* = 2
Mo *K*α radiation
μ = 0.09 mm^−1^
*T* = 293 (2) K
0.40 × 0.18 × 0.12 mm

#### Data collection

Oxford Diffraction Gemini S Ultra diffractometer
Absorption correction: multi-scan (*CrysAlis RED*; Oxford Diffraction, 2008 ▶) *T*_min_ = 0.964, *T*_max_ = 0.984
3205 measured reflections
1170 independent reflections
691 reflections with *I* > 2σ(*I*)
*R*_int_ = 0.067

#### Refinement

*R*[*F* ^2^ > 2σ(*F* ^2^)] = 0.045
*wR*(*F* ^2^) = 0.076
*S* = 0.89
1170 reflections
163 parameters
1 restraint
H-atom parameters constrained
Δρ_max_ = 0.15 e Å^−3^
Δρ_min_ = −0.14 e Å^−3^

### 

Data collection: *CrysAlis CCD* (Oxford Diffraction, 2008 ▶); cell refinement: *CrysAlis RED* (Oxford Diffraction, 2008 ▶); data reduction: *CrysAlis RED*; program(s) used to solve structure: *SHELXTL* (Sheldrick, 2008 ▶); program(s) used to refine structure: *SHELXTL*; molecular graphics: *ORTEP-3* (Farrugia, 1997 ▶); software used to prepare material for publication: *SHELXTL*.

## Supplementary Material

Crystal structure: contains datablocks I, global. DOI: 10.1107/S1600536808040105/wn2289sup1.cif

Structure factors: contains datablocks I. DOI: 10.1107/S1600536808040105/wn2289Isup2.hkl

Additional supplementary materials:  crystallographic information; 3D view; checkCIF report

## Figures and Tables

**Table 1:** Hydrogen-bond geometry (Å, °)

*D*—H⋯*A*	*D*—H	H⋯*A*	*D*⋯*A*	*D*—H⋯*A*
C15—H15*C*⋯O2^i^	0.96	2.54	3.301 (4)	136

Symmetry code: (i) 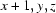.

**Fig. 1.**
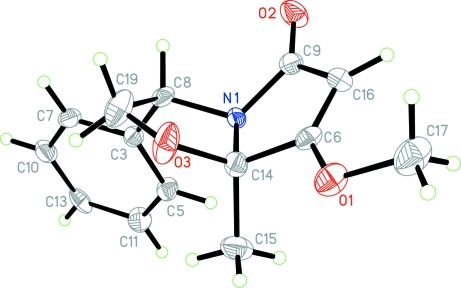
The molecular structure of the title compound with the atom-labelling scheme, showing 50% probability displacement ellipsoids. H atoms are drawn as spheres of arbitrary radius.

**Fig. 2.**
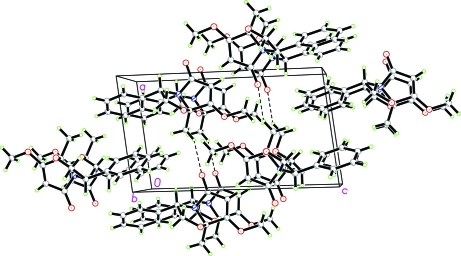
The packing of the molecules, viewed down the b axis. C—H···O hydrogen bond interactions are shown as dashed lines.

## supplementary crystallographic information

### Comment

The title compound, which was obtained by treating 5-hydroxy-1-((S)-2-hydroxy-1-phenylethyl) -4-methoxy-5-methyl-1H-pyrrol-2(5H)-one and picolinic acid with a catalytic amount of p-TsOH in CH_2_Cl_2_ at room temperature, is a key intermediate for the preparation of tetramic acids and methyl tetramates bearing C-5 methyl substituents; this is a key framework in a number of bioactive natural products, such as melophlins and mirabimide E (Huang & Deng, 2004; Huang et al., 2003; Jiang et al., 2009).

An X-ray crystal structure determination of the molecular structure of the title compound was carried out to determine its conformation. In the title chiral butterfly-like bicyclic lactam, C_14_H_15_NO_3_, in which the angle O3—C14—C6 is 112.3 (4)° and C8—N1—C9 is 119.6 (3)°, the phenyl and methyl groups are syn with respect to each other. The dihydropyrrrole ring adopts a boat conformation, whereas in the oxazole ring  the conformation is that of a slightly distorted boat. Bond lengths and angles are in agreement with values reported in the literature (Allen et al., 1987). The packing of molecules in the crystal structure is stabilized by intermolecular C—H···O hydrogen bonds.

### Experimental

To a cool (-78 °C) solution of (*S*)-1-(2-hydroxyl-1-phenylethyl) -3-methoxy-1*H*-pyrrole-2,5-dione(1.0 mmol) in anhydrous THF (10 ml) was added dropwise CH_3_MgBr (3.0 mmol) in diethyl ether under a nitrogen atmosphere. After stirring at the same temperature for 45 minutes, the reaction was quenched with saturated ammonium chloride (6 ml), and extracted with EtOAc (4 × 10 ml). The combined extracts were washed with brine, dried over Na_2_SO_4_, and concentrated under reduced pressure. The residue was purified by flash chromatography and yielded a mixture of diastereomers. To the mixture of diastereomers (0.51 mmol) in CH_2_Cl_2_ (10 ml) was added *p*-toluenesulfonic acid monohydrate (0.16 mmol.). After stirring for 30 minutes at room temperature, the mixture was quenched with saturated NaHCO_3_ solution. The organic layer was separated and the aqueous phase was extracted with CH_2_Cl_2_ (3 × 5 ml). The combined organic layers were washed with brine, dried over Na_2_SO_4_, and concentrated under reduced pressure. The residue was purified by flash chromatography to yield the title compound. Single crystals were obtained by slow evaporation of a petroleum ether / ethyl   acetate solution.

### Refinement

The hydrogen atoms were positioned geometrically, with C—H = 0.93, 0.98, 0.97 and 0.96 Å for phenyl, methine, methylene and methyl H atoms, respectively, and were included in the refinement in the riding model approximation. The displacement parameters of methyl H atoms were set to 1.5*U*_eq_(C), while those of other H atoms were set to 1.2*U*_eq_(C). In the absence of significant anomalous scattering effects, Friedel pairs were merged.

### Crystal data

**Table:**

C_14_H_15_NO_3_	*F*(000) = 260
*M**_r_* = 245.27	*D*_x_ = 1.295 Mg m^−^^3^
Monoclinic, *P*2_1_	Mo *K*α radiation, λ = 0.71073 Å
Hall symbol: P 2yb	Cell parameters from 672 reflections
*a* = 7.8238 (10) Å	θ = 2.9–32.6°
*b* = 5.9033 (7) Å	µ = 0.09 mm^−^^1^
*c* = 13.711 (3) Å	*T* = 293 K
β = 96.597 (14)°	Needle, colourless
*V* = 629.05 (16) Å^3^	0.40 × 0.18 × 0.12 mm
*Z* = 2	

### Data collection

**Table:**

Oxford Diffraction Gemini S Ultra diffractometer	1170 independent reflections
Radiation source: fine-focus sealed tube	691 reflections with *I* > 2σ(*I*)
graphite	*R*_int_ = 0.067
φ and ω scans	θ_max_ = 25.0°, θ_min_ = 2.9°
Absorption correction: multi-scan (*CrysAlis RED*; Oxford Diffraction, 2008)	*h* = −9→9
*T*_min_ = 0.964, *T*_max_ = 0.984	*k* = −4→7
3205 measured reflections	*l* = −16→14

### Refinement

**Table:**

Refinement on *F*^2^	Primary atom site location: structure-invariant direct methods
Least-squares matrix: full	Secondary atom site location: difference Fourier map
*R*[*F*^2^ > 2σ(*F*^2^)] = 0.045	Hydrogen site location: inferred from neighbouring sites
*wR*(*F*^2^) = 0.076	H-atom parameters constrained
*S* = 0.89	*w* = 1/[σ^2^(*F*_o_^2^) + (0.0229*P*)^2^] where *P* = (*F*_o_^2^ + 2*F*_c_^2^)/3
1170 reflections	(Δ/σ)_max_ = 0.016
163 parameters	Δρ_max_ = 0.16 e Å^−^^3^
1 restraint	Δρ_min_ = −0.14 e Å^−^^3^

### Special details

**Table:**

Geometry. All esds (except the esd in the dihedral angle between two l.s. planes) are estimated using the full covariance matrix. The cell esds are taken into account individually in the estimation of esds in distances, angles and torsion angles; correlations between esds in cell parameters are only used when they are defined by crystal symmetry. An approximate (isotropic) treatment of cell esds is used for estimating esds involving l.s. planes.
Refinement. Refinement of F^2^ against ALL reflections. The weighted R-factor wR and goodness of fit S are based on F^2^, conventional R-factors R are based on F, with F set to zero for negative F^2^. The threshold expression of F^2^ > 2sigma(F^2^) is used only for calculating R-factors(gt) etc. and is not relevant to the choice of reflections for refinement. R-factors based on F^2^ are statistically about twice as large as those based on F, and R- factors based on ALL data will be even larger.

### Fractional atomic coordinates and isotropic or equivalent isotropic displacement parameters (Å^2^)

**Table:**

	*x*	*y*	*z*	*U*_iso_*/*U*_eq_	
N1	0.1601 (4)	0.4015 (5)	0.7374 (3)	0.0243 (9)	
O2	−0.1216 (3)	0.4978 (5)	0.6847 (3)	0.0474 (10)	
C3	0.1854 (5)	0.2927 (6)	0.9142 (4)	0.0283 (12)	
O1	0.3713 (4)	0.3770 (5)	0.5217 (3)	0.0460 (9)	
C5	0.2722 (5)	0.4920 (7)	0.9416 (4)	0.0340 (12)	
H5A	0.2952	0.5946	0.8934	0.041*	
C6	0.2535 (5)	0.4025 (7)	0.5833 (4)	0.0323 (11)	
C7	0.1517 (5)	0.1443 (7)	0.9894 (4)	0.0340 (13)	
H7A	0.0933	0.0095	0.9740	0.041*	
C8	0.1279 (5)	0.2310 (7)	0.8097 (4)	0.0309 (12)	
H8A	0.0044	0.1977	0.8030	0.037*	
C9	0.0262 (5)	0.4666 (6)	0.6652 (4)	0.0310 (11)	
C10	0.2041 (5)	0.1961 (8)	1.0856 (5)	0.0416 (13)	
H10A	0.1807	0.0959	1.1347	0.050*	
C11	0.3251 (6)	0.5415 (8)	1.0386 (4)	0.0437 (14)	
H11A	0.3842	0.6753	1.0551	0.052*	
O3	0.3124 (4)	0.0915 (5)	0.6977 (3)	0.0601 (12)	
C13	0.2908 (5)	0.3940 (8)	1.1107 (4)	0.0418 (13)	
H13A	0.3259	0.4272	1.1762	0.050*	
C14	0.3068 (5)	0.3310 (7)	0.6872 (4)	0.0339 (12)	
C15	0.4755 (5)	0.4379 (9)	0.7300 (4)	0.0503 (14)	
H15A	0.5033	0.3885	0.7966	0.075*	
H15B	0.4645	0.5998	0.7284	0.075*	
H15C	0.5654	0.3929	0.6919	0.075*	
C16	0.0944 (5)	0.4851 (7)	0.5732 (4)	0.0386 (13)	
H16A	0.0370	0.5450	0.5158	0.046*	
C17	0.3234 (7)	0.4610 (10)	0.4231 (4)	0.0636 (16)	
H17A	0.4160	0.4352	0.3842	0.095*	
H17B	0.3001	0.6204	0.4255	0.095*	
H17C	0.2223	0.3830	0.3942	0.095*	
C19	0.2233 (7)	0.0254 (7)	0.7733 (5)	0.0562 (17)	
H19A	0.3023	−0.0355	0.8266	0.067*	
H19B	0.1414	−0.0921	0.7509	0.067*	

### Atomic displacement parameters (Å^2^)

**Table:**

	*U*^11^	*U*^22^	*U*^33^	*U*^12^	*U*^13^	*U*^23^
N1	0.0233 (17)	0.0272 (18)	0.022 (3)	0.0013 (15)	0.0014 (17)	0.0048 (18)
O2	0.0300 (16)	0.069 (2)	0.043 (3)	0.0111 (17)	0.0055 (16)	0.0099 (19)
C3	0.026 (2)	0.026 (3)	0.034 (4)	0.0031 (18)	0.005 (2)	0.002 (2)
O1	0.0494 (19)	0.0528 (19)	0.039 (3)	−0.0044 (17)	0.0195 (19)	−0.003 (2)
C5	0.042 (2)	0.023 (2)	0.037 (4)	−0.001 (2)	0.002 (2)	−0.003 (3)
C6	0.036 (3)	0.029 (2)	0.033 (3)	−0.012 (2)	0.007 (2)	−0.005 (2)
C7	0.027 (2)	0.029 (3)	0.046 (4)	−0.0049 (18)	0.004 (3)	−0.001 (3)
C8	0.034 (2)	0.025 (2)	0.033 (4)	−0.011 (2)	0.001 (2)	−0.003 (2)
C9	0.029 (2)	0.030 (2)	0.034 (3)	−0.002 (2)	0.004 (2)	0.000 (2)
C10	0.035 (3)	0.052 (3)	0.036 (4)	0.003 (3)	−0.001 (3)	0.012 (3)
C11	0.054 (3)	0.036 (3)	0.039 (4)	−0.005 (2)	−0.008 (3)	−0.004 (3)
O3	0.068 (2)	0.035 (2)	0.086 (4)	0.0224 (16)	0.047 (2)	0.020 (2)
C13	0.041 (3)	0.058 (3)	0.023 (3)	0.013 (3)	−0.009 (2)	0.004 (3)
C14	0.029 (2)	0.030 (3)	0.044 (4)	0.010 (2)	0.011 (2)	0.006 (2)
C15	0.030 (2)	0.078 (4)	0.043 (4)	−0.004 (3)	0.003 (2)	0.004 (3)
C16	0.030 (2)	0.042 (3)	0.041 (4)	−0.001 (2)	−0.005 (2)	0.007 (3)
C17	0.079 (4)	0.074 (4)	0.040 (4)	−0.025 (3)	0.018 (3)	−0.004 (4)
C19	0.082 (4)	0.032 (3)	0.059 (5)	−0.006 (3)	0.024 (4)	−0.003 (3)

### Geometric parameters (Å, °)

**Table:**

N1—C9	1.410 (5)	C10—C13	1.374 (7)
N1—C8	1.455 (5)	C10—H10A	0.9300
N1—C14	1.465 (5)	C11—C13	1.366 (7)
O2—C9	1.230 (4)	C11—H11A	0.9300
C3—C5	1.389 (5)	O3—C19	1.371 (6)
C3—C7	1.402 (6)	O3—C14	1.421 (5)
C3—C8	1.497 (7)	C13—H13A	0.9300
O1—C6	1.329 (5)	C14—C15	1.519 (6)
O1—C17	1.448 (7)	C15—H15A	0.9600
C5—C11	1.378 (7)	C15—H15B	0.9600
C5—H5A	0.9300	C15—H15C	0.9600
C6—C16	1.329 (5)	C16—H16A	0.9300
C6—C14	1.498 (7)	C17—H17A	0.9600
C7—C10	1.369 (7)	C17—H17B	0.9600
C7—H7A	0.9300	C17—H17C	0.9600
C8—C19	1.538 (6)	C19—H19A	0.9700
C8—H8A	0.9800	C19—H19B	0.9700
C9—C16	1.429 (6)		
			
C9—N1—C8	119.6 (3)	C19—O3—C14	110.3 (4)
C9—N1—C14	107.9 (4)	C11—C13—C10	119.5 (5)
C8—N1—C14	109.3 (3)	C11—C13—H13A	120.2
C5—C3—C7	117.2 (5)	C10—C13—H13A	120.2
C5—C3—C8	123.4 (4)	O3—C14—N1	104.6 (3)
C7—C3—C8	119.5 (4)	O3—C14—C6	112.3 (4)
C6—O1—C17	115.5 (4)	N1—C14—C6	102.6 (3)
C11—C5—C3	121.7 (5)	O3—C14—C15	111.0 (4)
C11—C5—H5A	119.2	N1—C14—C15	113.2 (4)
C3—C5—H5A	119.2	C6—C14—C15	112.6 (4)
O1—C6—C16	133.2 (5)	C14—C15—H15A	109.5
O1—C6—C14	115.8 (4)	C14—C15—H15B	109.5
C16—C6—C14	111.0 (4)	H15A—C15—H15B	109.5
C10—C7—C3	120.6 (4)	C14—C15—H15C	109.5
C10—C7—H7A	119.7	H15A—C15—H15C	109.5
C3—C7—H7A	119.7	H15B—C15—H15C	109.5
N1—C8—C3	115.3 (3)	C6—C16—C9	108.7 (5)
N1—C8—C19	101.3 (4)	C6—C16—H16A	125.7
C3—C8—C19	113.5 (4)	C9—C16—H16A	125.7
N1—C8—H8A	108.8	O1—C17—H17A	109.5
C3—C8—H8A	108.8	O1—C17—H17B	109.5
C19—C8—H8A	108.8	H17A—C17—H17B	109.5
O2—C9—N1	122.0 (4)	O1—C17—H17C	109.5
O2—C9—C16	129.5 (5)	H17A—C17—H17C	109.5
N1—C9—C16	108.5 (3)	H17B—C17—H17C	109.5
C7—C10—C13	121.0 (5)	O3—C19—C8	109.2 (4)
C7—C10—H10A	119.5	O3—C19—H19A	109.8
C13—C10—H10A	119.5	C8—C19—H19A	109.8
C13—C11—C5	120.0 (5)	O3—C19—H19B	109.8
C13—C11—H11A	120.0	C8—C19—H19B	109.8
C5—C11—H11A	120.0	H19A—C19—H19B	108.3
			
C7—C3—C5—C11	0.7 (5)	C19—O3—C14—N1	−19.4 (6)
C8—C3—C5—C11	−179.1 (4)	C19—O3—C14—C6	−129.9 (5)
C17—O1—C6—C16	2.4 (7)	C19—O3—C14—C15	103.0 (5)
C17—O1—C6—C14	−175.9 (4)	C9—N1—C14—O3	−107.5 (4)
C5—C3—C7—C10	−0.3 (6)	C8—N1—C14—O3	24.0 (5)
C8—C3—C7—C10	179.5 (4)	C9—N1—C14—C6	9.9 (4)
C9—N1—C8—C3	−130.6 (4)	C8—N1—C14—C6	141.4 (3)
C14—N1—C8—C3	104.4 (4)	C9—N1—C14—C15	131.5 (4)
C9—N1—C8—C19	106.3 (4)	C8—N1—C14—C15	−97.0 (4)
C14—N1—C8—C19	−18.6 (5)	O1—C6—C14—O3	−74.1 (5)
C5—C3—C8—N1	−3.5 (6)	C16—C6—C14—O3	107.3 (4)
C7—C3—C8—N1	176.7 (3)	O1—C6—C14—N1	174.2 (3)
C5—C3—C8—C19	112.8 (4)	C16—C6—C14—N1	−4.4 (5)
C7—C3—C8—C19	−67.0 (5)	O1—C6—C14—C15	52.1 (5)
C8—N1—C9—O2	42.2 (6)	C16—C6—C14—C15	−126.5 (4)
C14—N1—C9—O2	167.8 (4)	O1—C6—C16—C9	179.0 (4)
C8—N1—C9—C16	−137.6 (4)	C14—C6—C16—C9	−2.7 (5)
C14—N1—C9—C16	−12.0 (4)	O2—C9—C16—C6	−170.5 (4)
C3—C7—C10—C13	−0.1 (6)	N1—C9—C16—C6	9.2 (5)
C3—C5—C11—C13	−0.7 (6)	C14—O3—C19—C8	8.1 (6)
C5—C11—C13—C10	0.3 (6)	N1—C8—C19—O3	6.7 (5)
C7—C10—C13—C11	0.1 (6)	C3—C8—C19—O3	−117.5 (5)

### Hydrogen-bond geometry (Å, °)

**Table:**

*D*—H···*A*	*D*—H	H···*A*	*D*···*A*	*D*—H···*A*
C15—H15C···O2^i^	0.96	2.54	3.301 (4)	136

Symmetry codes: (i) *x*+1, *y*, *z*.
